# Industrially Important Fungal Carotenoids: Advancements in Biotechnological Production and Extraction

**DOI:** 10.3390/jof9050578

**Published:** 2023-05-16

**Authors:** Tahira Naz, Samee Ullah, Yusuf Nazir, Shaoqi Li, Bushra Iqbal, Qing Liu, Hassan Mohamed, Yuanda Song

**Affiliations:** 1 Colin Ratledge Center for Microbial Lipids, College of Agricultural Engineering and Food Science, Shandong University of Technology, Zibo 255000, China; tahiranaz@sdut.edu.cn (T.N.); samee1264@gmail.com (S.U.); yusufnazir91@yahoo.com (Y.N.); lsq163947@126.com (S.L.); bushra.iqbal1212@gmail.com (B.I.); qingliu0906@sdut.edu.cn (Q.L.); 2 Faculty of Allied Health Sciences, University Institute of Food Science and Technology, The University of Lahore, Lahore 54000, Pakistan; 3 Department of Food Sciences, Faculty of Science and Technology, Universiti Kebangsaan Malaysia, Bangi 43600, Malaysia; 4 Innovation Centre for Confectionery Technology (MANIS), Faculty of Science and Technology, Universiti Kebangsaan Malaysia, Bangi 43600, Malaysia; 5 Department of Botany and Microbiology, Faculty of Science, Al-Azhar University, Assiut 71524, Egypt

**Keywords:** fungal carotenoids, genetic modification, biosynthetic pathways, extraction

## Abstract

Carotenoids are lipid-soluble compounds that are present in nature, including plants and microorganisms such as fungi, certain bacteria, and algae. In fungi, they are widely present in almost all taxonomic classifications. Fungal carotenoids have gained special attention due to their biochemistry and the genetics of their synthetic pathway. The antioxidant potential of carotenoids may help fungi survive longer in their natural environment. Carotenoids may be produced in greater quantities using biotechnological methods than by chemical synthesis or plant extraction. The initial focus of this review is on industrially important carotenoids in the most advanced fungal and yeast strains, with a brief description of their taxonomic classification. Biotechnology has long been regarded as the most suitable alternative way of producing natural pigment from microbes due to their immense capacity to accumulate these pigments. So, this review mainly presents the recent progress in the genetic modification of native and non-native producers to modify the carotenoid biosynthetic pathway for enhanced carotenoid production, as well as factors affecting carotenoid biosynthesis in fungal strains and yeast, and proposes various extraction methods to obtain high yields of carotenoids in an attempt to find suitable greener extraction methods. Finally, a brief description of the challenges regarding the commercialization of these fungal carotenoids and the solution is also given.

## 1. Introduction

Carotenoids are a type of naturally occurring lipophilic molecules, with the majority being C40 terpenoids. They act as membrane-protective antioxidants by scavenging oxygen and peroxyl radicals. Photosynthetic organisms such as algae, cyanobacteria, and plants, as well as some fungi and bacteria, commonly synthesize carotenoids. These pigments are also frequently used as food colorants [1]. Most animals are unable to produce carotenoids themselves, but they can obtain them through their diet and modify them structurally. However, some invertebrate animals, such as *Ascidia purpurea*, hemipteran (aphids, adelgids, phylloxerids), and dipteran (gall midges) insects and mites, have shown the ability to synthesize carotenoids de novo [2]. Although carotenoids are extensively distributed in plants, they have low cellular carotenoid content. Additionally, large cultivation areas, specific agricultural techniques, seasonal and geographical variations, raw material costs, and sometimes manual harvests contribute to the high cost of plant-sourced carotenoids [3]. Fortunately, various microorganisms are capable of producing carotenoids, although not all of them are commercially or economically viable. Microbial products offer several advantages, such as immediate availability throughout the year, low environmental impact, lower production costs compared to plant extraction, high yield, and extensive development prospects [3,4].

Carotenoid production in fungi is not essential for its growth in contrast to photoautotrophic organisms, thus accumulating in considerably lesser amounts compared to algae and plants [5]. Fungal carotenoids, however, offer a structural variety not present in other sources of carotenoids. Various carotenoids of commercial interest have been produced in many fungal species using metabolic and classical engineering techniques, which are presented in this review. The review offers up-to-date information on advancements in the biotechnological production of industrially important fungal carotenoids. Initially, classifications, structures, and the potential applications of industrially important fungal carotenoids are discussed. The effects of cultivation conditions on the biotechnological production of fungal carotenoids are also discussed. The emphasis is on the recent biotechnological production of fungal and yeast carotenoids using modern genetic engineering techniques. Moreover, the latest extraction procedures are also discussed to find a greener solution for carotenoid extraction.

### Carotenoids Classification

**Figure 1:**
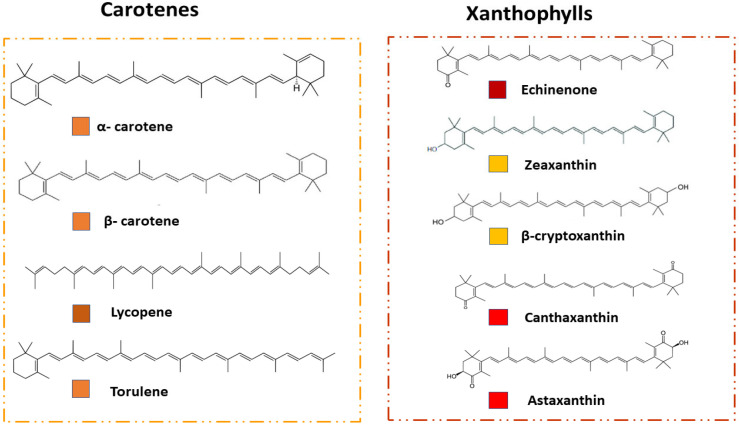
Chemical structure of a few major carotenes and xanthophylls.

Carotenoids classification can be conducted on the basis of their chemical and nutritional characteristics. Chemically, they are categorized into two main classes such as carotenes and xanthophylls. Carotenes is a well-renowned group that contains carbon (C) and hydrogen (H) in the chemical structure, e.g., α-carotene, β-carotene, γ-carotene, torulene, and lycopene, while the xanthophylls is the second class, which contains C, H, and oxygen (O) in their chemical structure, e.g., zeaxanthin, lutein, torularhodin, canthaxanthin, and astaxanthin [6]. According to their nutritional properties, carotenoids are normally classified as pro-vitamin A (α-carotene, β-carotene, β-cryptoxanthin), or non-provitamin A (zeaxanthin, lycopene, lutein) [7,8], and ketocarotenoids, e.g., canthaxanthin and astaxanthin [9]. The structural characteristics of carotenoids, such as the presence of two terminal rings connected by a double conjugated chain or polyene system (such as astaxanthin), are thought to contribute to their antioxidant capability. Carotenoids are usually composed of eight isoprene units, including conjugated double bonds, which helps them to absorb light and give them their distinctive yellow, orange, or reddish hues [10]. Figure 1 depicts the chemical structure of industrially important carotenoids of each class.

## 2. Potential Applications and Biological Functions of Carotenoids

Carotenoids are valuable molecules due to their ability to both provide color and act as antioxidants. As a result, researchers and industries have taken a keen interest in their various applications across many fields [11]. The most crucial and valuable carotenoids are β-carotene, lycopene, lutein, canthaxanthin, zeaxanthin, and astaxanthin, which are widely utilized in the food, feed, and cosmetic industries (as outlined in Table 1). However, it is worth noting that the regulations governing their use differ by country, with each specifying the source, purity, and amounts of the colorants allowed [12].

The immune system-boosting effect of these carotenoids are due to their antioxidant capability and ultimately aid in preventing different kinds of cancers, both in humans and animals [13], by reducing the toxic activity of reactive oxygen species (ROS). These carotenoids are suggested to be involved in strengthening the immune system by boosting lymphocyte blastogenesis, raising the population of particular subsets of lymphocyte, increasing the cytotoxic role of lymphocyte, and promoting the synthesis of a number of cytokines [14]. Being a vitamin A precursor, carotenoids may serve as a bioactive compound in the reduction of degenerative diseases such as cardiovascular diseases, muscular degeneration, cataracts, and cancer [15]. The human diet contains carotenoids in the form of lutein, β-cryptoxanthin, zeaxanthin, α- carotene, β-carotene, and lycopene [16]. Individuals with lutein and zeaxanthin consumption in their diet are reported to have a reduced risk of breast cancer and a lower frequency of ocular problems [11]. Consumption of lycopene has shown many health benefits due to its high antioxidant potential, which reduces the risk of prostate cancer and cardiac failure [17]. Intake of food rich in β-carotene was reported to reduce the risk of cardiovascular diseases, which is one of the leading causes of death in the world. Foods fortified with β-carotene have shown protection against esophageal cancer [12]. Another study has also suggested that carotenoids from *Neochloris oleoabundans* can inhibit the proliferation of colon cancer cells, allowing their use as functional food additives or nutraceuticals with the ability to prevent colon cancer [18]. Similarly, β-carotene, capsanthin, and astaxanthin possess antiproliferative effects on leukemic K562 cells [19]. However, the roles of these carotenoids are controversial, and there are only a few studies [20]. Hence, more studies are required to fully establish the beneficial role of these carotenoids in human and animal health.

**Table 1:** Major useful fungal carotenoids, applications and their biological activities [21].

Carotenoids	Application	Activity	Reference
Astaxanthin	Fish feed and cosmetic industry	Antioxidant, photo-protectant, anti-inflammatory and anticancer.	[22]
β-carotene	Animal feed, Nutraceutical and cosmetics industries	Antioxidant, anticancer, and vitamin A precursor	[23]
Canthaxanthin	Cosmetic, Poultry and fish feed	Antioxidant and anticancer	[24]
Lutein	Poultry feed, functional nutrient	Antioxidant	[25]
Lycopene	Supplements in functional food, additives in cosmetics	Antioxidant and anticancer	[26]
Neurosporaxanthin	Feed or food additive	Antioxidant	[27]
Torulene and torularhodin	Feedstock, food, and cosmetic additives	Antioxidant, and anticancer	[28]

## 3. Industrially Important Fungal Carotenoids

Fungi have the ability to biosynthesize a diverse range of carotenoids and have garnered attention due to their biochemistry, structural diversity, and genetic production. Examples of carotenoids commonly produced in fungi include β-carotene, lycopene, canthaxanthin, neurosporaxanthin, and astaxanthin. These pigments are synthesized by fungi for various non-essential roles such as stress mediation and the production of physiologically active by-products such as trisporoids and apocarotenoid derivatives (trisporic acid). The antioxidant potential of carotenoids can aid in the survival of fungi in their natural environment, protecting fungal cells against stresses such as oxidative and UV or visible light exposure, as observed by several researchers [29]. In addition to their protective functions, carotenoids also play a critical function in the mating process of mucoraceous fungi [30].

Carotenoids are produced by nearly all fungal species, including non-carotenogenic species that have undergone genetic manipulation. Classical and metabolic engineering strategies have been established to enhance the concentrations of economically useful carotenoids in diverse fungal species to match those observed in other organisms [31]. However, less is known about the production of xanthophylls in fungi, and researchers have attempted to produce these carotenoids in fungal species using genetic engineering techniques in recent years [32]. Fungi may serve as suitable heterologous hosts for the production of these valuable carotenoids if the genes and enzymes involved in the pathway are fully understood. Notably, fungi have been observed to produce more carotenoids than other investigated heterologous hosts, such as archaebacteria, Escherichia coli, other eubacteria, and plants [33]. Fungi can be served as an ideal model organism for carotenogenic studies and for the determination of various gene functions in eukaryotes. Additionally, the synthesis of carotenoids in several fungi is regulated by light [34]. A summary of the carotenoid distribution in various fungal classes is provided in Table 2.

Mucoraceae species typically contain bicyclic β-carotene. β-carotene was also discovered in a few other Ascomycota and Basidiomycota fungi as the final product, but the majority of carotenoids in both phyla were oxygenated derivatives. For example, two native carotenogenic Mucormycotina fungi, *Phycomyces blakesleeanus*, and *Blakeslea trispora* are well-recognized model fungi for fermentative β-carotene production at commercial scales [45]. In Chytridiomycota and Zygomycota, β-carotene or γ-carotene are accumulated as the main carotenoids, while in species of Blastocladiale, γ-carotene acts as the end product of carotenoid synthesis, as in the case of *Allomyces* and *Blastocladiella* species, along with lower levels of β-carotene. Other intermediates of the carotenoid synthesis pathway can be found in a variety of species, depending on the strains and their cultural conditions. The carotenoid composition of most Ascomycotina fungi is similar to Chytridiomycota and Zygomycota. However, the orders Sphaeriales and Pezizales may also include unique carotenoids [5]. Regarding yeast, asporous yeasts are well known for their yellowish reddish color, which is attributed to the accumulation of carotenoids. For example, *Xanthophyllomyces dendrorhous* is among the few fungi that can synthesize astaxanthin along with the production of other 3-hydroxy and 4-keto derivatives [46].

In the next sections, industrially important carotenoids in fungal and yeast strains will be discussed in detail.

β-carotene has been reported as the most dominant carotenoid in Zygomycetes of order Mucorales, such as *B. trispora* [47], *P. blakesleeanus*, and *M. circinelloides* [45]. In various fungal species, β-carotene works as an intermediate in the biosynthesis of other carotenoids and is found in higher concentrations in mutants of *X. dendrorhous* or *R. glutinis* [1]. Industrially, it is important due to its provitamin A activity, antioxidant potential, and immunomodulatory action, and its preventive role against various cancers and cardiovascular diseases (CVD) [47].

Torulene and torularhodin are other fascinating pigments reported to be produced only by fungi and yeasts. *Rhodotorula* and *Sporobolomyces* are the most prominent producers of the above-mentioned carotenoids [28]. As precursors of vitamin A, as well as having antioxidant, anti-ageing, and anticancer activities, and a role in boosting immunity, these carotenoids are of specific interest for industrial applications. Compared to β-carotene, torulene and torularhodin have a longer polyene chain and one β-ionone ring. Additionally, torularhodin exhibits significant antioxidant activity and is one of the few carotenoids that has a carboxylic acid function, meaning it could be used for feedstock, food, and cosmetic additives. However, the production of some carotenoids, such as zeaxanthin, is still in the early stages of development due to their relatively low production rate [48], especially compared to nutraceutical carotenoids such as lycopene, β-carotene, and astaxanthin [49]. To produce these two pigments on a commercial scale, highly efficient yeast strains are necessary, which can be achieved through various strain improvement approaches such as mutagenesis and the optimization of cultivation conditions [23].

Neurosporaxanthin is another valuable carboxylic colored compound produced in a few filamentous fungi such as species of the genera *Neurospora* and *Fusarium*. It was first reported in *N. crassa* as an acidic carotenoid that was mixed with other carotenoids, and it imparts *N. crassa* cultures, which have a typical orange coloring. Neurosporaxanthin is not a common naturally-present carotenoid; in addition to *Neurospora* and *Fusarium*, only a few species of fungi, including *Verticillium* [48] and *Podospora* [49], have been reported to contain this carotenoid [27]. The initial steps of β-carotene and astaxanthin synthesis are reported to coincide with those of Neurosporaxanthin, beginning with the production of phytoene from geranylgeranyl pyrophosphate GGPP. The neurosporaxanthin synthetic pathway in reported fungi has a few distinctive steps. Firstly, there are five desaturations rather than four, and there is only one cyclization step. The intermediates of *N. crassa* and *F. fujikuroi* can vary based on particular carotenoids in which the β-ionone rings are inserted [29]. The antioxidant capabilities of this substance have not been studied because it is not part of the human diet. The potential role of neurosporaxanthin as a protectant against oxidative damage in fungi has been indicated in *Fusarium* and *Neurospora*, as reported by Ligusa et al. [50], who observed the light-induced synthesis of neurosporaxanthin in *F. aquaeductuum* and *N. crassa* when grown in media containing hydrogen peroxide (H_2_0_2_). More recent research investigated its antioxidant activity, and these analytical experiments showed that neurosporaxanthin-rich extracts had stronger antioxidant capacity than neurosporaxanthin-deficient extracts in terms of quenching activity and scavenging activity [27].

**Figure 2:**
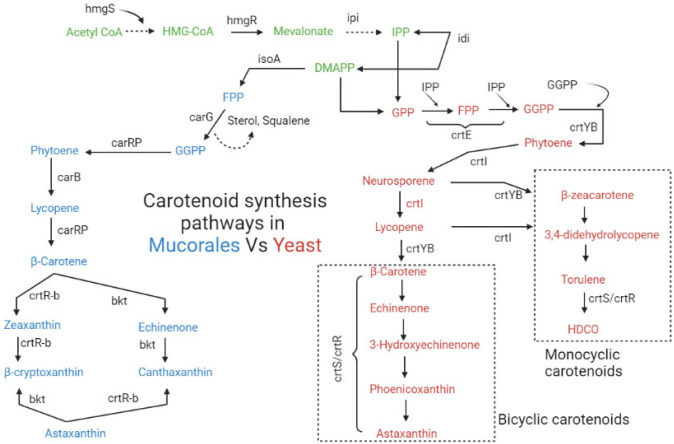
Biosynthesis pathway for astaxanthin production in *Mucor* and *X. dendrorhous*. Green fonts show the similar precursors and genes involved in initial isoprenoid biosynthesis pathway of both fungi and yeast. The left side of the image with blue fonts represents the proposed astaxanthin biosynthesis pathway in *Mucor* and the right side with red fonts represents the carotenoid biosynthesis pathway in red yeast *X. dendrorhous*.

Canthaxanthin is a red keto-carotenoid that is naturally found in only one fungal genus, the basidiomycete *Cantharellus* [41]. Canthaxanthin is synthesized as an intermediate carotenoid in the β-carotene to the astaxanthin biosynthetic pathway (Figure 2). It possesses greater antioxidant ability compared to β-carotene [51], gaining more commercially significant importance with extensive applications in industries [52]. For instance, canthaxanthin is employed as a feed additive in the poultry industry for the red color in egg yolks and skins [53], and in the cosmetics industry as a pigmenting agent.

Astaxanthin is another valuable carotenoid that is only produced by *X. denrorhous*, a basidiomycetous yeast [54]. This molecule is synthesized in the mentioned yeast by adding hydroxyl and keto groups to rings of the β-carotene. Separate hydroxylase and ketolase are involved in the synthesis of astaxanthin from β-carotene in bacteria and algae. However, in *X. dendrorhous*, a single bifunctional enzyme of oxygenase P450 appears to carry out both enzymatic activities, at least in part with the assistance of a cytochrome P450 [55]. It exists in three different stereoisomers: (3*S*, 3′*S*), (3*R*, 3′*S*)), and (3′*R*, 3*R*). All these forms are present in natural sources. Astaxanthin has higher polarity and antioxidant potential in comparison to other carotenoids such as zeaxanthin, lutein, canthaxanthin, and β-carotene, which is accredited to its special 3-hydroxyl and 4-keto functional groups [56]. It has 550 times more antioxidant power compared to vitamin E, and is 6000 times higher than vitamin C. Recent studies have demonstrated many beneficial characteristics of astaxanthin on human health such as anti-diabetic, anti-inflammatory, antibacterial, antitumor, neuroprotective, photoprotective, immunostimulant, and its beneficial effect on cardiovascular health [29,57].

## 4. Carotenoid Biosynthesis Pathway in Fungi

Fungal carotenoids are produced through the mevalonic acid (MVA) pathway located in the cytosol, and complex genetic mechanisms are involved in the regulation of carotenoid synthesis. In Fungal cells, carotenoids are derived from 3-hydroxy-3-methyl glutaryl-CoA (HMG-CoA), which acts as a first substrate for the biosynthesis of mevalonate. This HMG-CoA is produced from acetoacetyl-CoA by the catalytic activity of HMG-CoA synthase (*hmgS*) and serves as the first precursor for the synthesis of mevalonate. HMG-CoA is converted into mevalonate by catalytic reaction of HMG-CoA reductase (*hmgR*). Fungal Isopentenyl pyrophosphate (IPP, C_5_) is produced from mevalonate and serves as a building block to all terpenoid synthesis. IPP is not reactive enough to start a condensation reaction to high terpenoids and isomerized to dimethylallyl pyrophosphate (DMAP) by the IPP isomerase (*ipi*). IPP and DMAP then further proceed through condensation to form intermediates farnesyl pyrophosphate FPP and geranyl pyrophosphate GPP, and then the carbon chain is elongated with further IPP units. These steps are controlled by farnesyl pyrophosphate (FPP, C_15_) and geranylgeranyl pyrophosphate (GGPP, C_20_) synthases (*carG*), respectively. These intermediates may then undergo self-condensation to C_30_ and C_40_ precursors of sterols and carotenoids, respectively. This conversion is considered as the first bottleneck in the mevalonate pathway since *carG* was found to be co-regulated with the structural genes (*carB* and *carRP*) of the carotenoid biosynthesis pathway [58].

The first committed step of carotenoid synthesis starts with the condensation of two C_20_ GGPP units. These two molecules of GGPP are converted into phytoene and this reaction is catalyzed by phytoene synthase (*crtB*). Then, phytoene dehydrogenase carries out many dehydrogenations reactions to produce phytofluene, neurosporene, and lycopene [28]. The cyclization of lycopene, a precursor to the cyclic carotenoids, produces β-carotene, γ-carotene, echinenone, torulene, torularhodin, and astaxanthin [59,60]. In *Mucorales* two enzymes, Phytoene dehydrogenase *carB* [34] and a bifunctional enzyme known as phytoene synthase/lycopene cyclase *carRP* [61], convert phytoene to lycopene and then from lycopene to β-carotene, respectively (Figure 2). These two structural genes *carB* and *carRP* from *M. circinelloides* were overexpressed in a heterologous way in *Y. lipolytica* to improve the β-carotene production [62,63]. Genetic analysis of carotene mutation in *P. blakesleeanus* found that only two structural genes, *carB* and *carR*, are responsible for the conversion of phytoene to β-carotene [64,65].

Other than pathways to β-carotene, genes and enzymes responsible for neurosporaxanthin and astaxanthin synthesis have not yet been described fully in fungi. However, hypothetical reaction pathways to introduce oxy groups into the carotenoid structure can be expected using analogies involved in bacterial carotenogenesis. For instance, it has been observed that accumulated carotenoids in fungi, i.e., β-carotene, can act as a key intermediate for the synthesis of hydroxylated or oxygenated derivatives of β-carotene, such as zeaxanthin, echinenone canthaxanthin, and astaxanthin, in fungi and yeasts [32]. Commonly, two important enzymes that accept several substrates are involved in the conversion of β-carotene into its keto derivatives compounds and have been reported in microalgae [66] and yeasts [39,67]: (1) β-carotene ketolase, which is responsible for the conversion of β-carotene to canthaxanthin and from zeaxanthin to astaxanthin by introducing the keto group at position C-4 of the ring structure; (2) β-carotene hydroxylase adds the hydroxyl group in β-carotene at position C-3, converting it into zeaxanthin [68], and can also convert canthaxanthin to astaxanthin. The proposed mechanism of canthaxanthin and astaxanthin biosynthesis in species of *Mucorales* is shown in Figure 2. Since these two enzymes are rate limiting enzymes for xanthophylls production, many researchers have targeted the genes coding these enzymes for the improved production of valuable carotenoids such as canthaxanthin and astaxanthin [31,69].

In *X. dendrorhous*, the desaturase *crtI* and phytoene synthase/desaturase *crtYB* carry out the metabolic pathways leading to β-carotene. Verdoes et al. [70] carried out the functional investigation of the *crtYB* homolog from *X. dendrorhous* and showed that the expressed gene product catalyzed both phytoene production and lycopene cyclisation, while for astaxanthin, a unique gene astaxanthin synthase *crtS* belonging to the P450 oxygenase family with a single bifunctional enzyme function has been described in this yeast. This gene *crtS* interacts with cytochrome P450 reductase *crtR* to carry out the conversion of β-carotene into astaxanthin in this red yeast [55,71], as shown in Figure 2. With the documented roles of these two genes in astaxanthin biosynthesis, many researchers have targeted the mentioned genes for the genetic modification of yeast and fungi to obtain high astaxanthin-producing strains. For example, the overexpression of *crtS* resulted in the up-regulation of synthesis-related genes with improved astaxanthin production [72]. Moreover, this gene was expressed in a heterologous way in the fungus *M. circinelloides*, improving its ability to synthesize zeaxanthin and β-cryptoxanthin [73].

## 5. Cultivation Condition Affecting Biotechnological Production of Carotenoids

The cultivation parameters must be improved to increase production when it comes to manufacturing for industrial applications. The growing conditions are also crucial for the extraction because they can alter the cell structure [74]. The process of carotenoid synthesis in fungi can be significantly influenced by various cultural conditions. Carotenoids, which serve crucial biological functions such as safeguarding cells against oxidative stress and enhancing photosynthesis, necessitate specific cultural conditions, i.e., correct temperature, pH, light, and nutrition availability, among other factors to achieve high production levels. For instance, some fungi may produce more carotenoids under higher temperatures, while others may require the absence of light. Furthermore, the growth medium composition is very important in carotenoid production, with particular nutrients, including nitrogen and carbon sources, vitamins, and minerals, being essential. Consequently, by manipulating these cultural conditions, it is feasible to increase the carotenoid products from fungi.

### 5.1. pH

The production of carotenoids during fermentation is influenced by the pH level of the environment, which affects both cell growth and the biosynthesis of these pigments. Throughout the fermentation process, the pH changes and these changes are linked to the growth of microorganisms. Usually, there is a decrease in pH during the initial 72 h of fermentation, followed by a pH spike during a peak phase of carotenoid production. Towards the end of the carotenoid production process, the pH levels stabilize and remain constant [15]. According to research, *R. glutinis* removed from *Scabiosa atropurpura* has grown well at an optimum of pH 6.2 with 12.5 g/L sucrose and 0.1 g/L zinc sulphate in cultivation medium, over the course of six days at 25 °C. Most carotenoid syntheses took place in the stationary phase, with the greatest level (861 µg/g) of total carotenoids being reported after five days [75].

On the other hand, Hernández-Almanza et al. [76] found that the optimal pH for growing *R. glutinis* was close to 4.0, and the total carotenoid concentration was 340 µg/mL under these conditions, while other researchers reported the peak astaxanthin production (1.252 mg/g) from *X. dendrorhous* at pH 5 while keeping the agitation and temperature at 300 RPM and 20 °C, respectively [77]. Similarly, other researchers reported the optimum pH 5.91 for total carotenoids (3.48 μg/mL) while growing the *R. glutinis* under a different pH range from 3.0 to 7.5 [78]. β-carotene production is also affected by the change in pH in *Saccharomyces cerevisiae* growing on the YNB media with a maximum β-carotene yield 16.8 ± 1.8 mg/g, reported at pH 4, although no significant effect of pH was seen when under the same conditions, *S. cerevisiae* was grown on the YPD media [79].

Two studies on *R. rubra* yeast reported pH 5.0 as optimum for carotenoid production [80], while 6.0–7.0 pH was reported for the production of torularhodin [81]. Moreover, these authors also observed that cell growth was limited at pH 8.0 and carotenoid production was not favorable at pH 3.0. Similarly, pH 5.85 was reported for the highest β-carotene production, i.e., 262.12 mg/L from the mutant species of *R. acheniorum* [82]. Several other investigations have explored how pH affects the synthesis of carotenoids in various fungal species. Generally, the best pH for carotenoid synthesis depends on the specific fungal species and the chosen growth medium.

### 5.2. Light

The intensity of light has a major role in carotenoid synthesis but accumulation can vary among different microorganisms. In some cases, an increase in light intensity can result in improved microbial growth, but this does not necessarily lead to an increase in carotenoid accumulation. However, while light intensity can be an effective tool in stimulating carotenoid biosynthesis, the methods and results vary in different species and it is important to consider the relationship between light intensity, microbial growth, and enzyme activity in order to optimize carotenoid production [15]. In other studies, it is also reported that the pigment production in fungi is often caused by environmental stressors, such as light radiation, oxidation, lack of nutrients, high salt concentrations, and host immunity, and ultimately, rather than producing primary metabolites for cell growth, fungi switch to producing secondary metabolites in the form of pigments [83]. This is thought to be a defense mechanism, protecting the fungi’s mycelia from degradation by enzymes produced by other species. The yield of pigments is believed to stem from the fungi’s need to protect themselves from these environmental stressors [83]. For instance, in the case of torularhodin synthesis in yeast cells, it was found that exposure to weak white light led to an increase in torularhodin synthesis [84]. It is also reported that exposure to white light increased the β-carotene accumulation in *R. glutinis* during its logarithmic growth phase [85]. Two strains of *M. circinelloides* also showed an increase in the β-carotene synthesis when exposed to continuous light. A 2.7-fold increase in β-carotene synthesis was recorded in the *M. circinelloides* CBS 277.49 with the yield of 698.4 μg/g, while in *M. circinelloides* strain WJ11, the increase was 2.2-fold with a yield of 275 μg/g of β-carotene [13]. The photo-inducibility of astaxanthin synthesis in *X. dendrorhous* has also been well established through experiments [59]. Conversely, *X. dendrorhous* has been shown to reduce astaxanthin production from 969 μg/g to 930 μg/g while growing under light conditions. However, to attain the maximum concentration of 930 μg/g, it takes 7 days, while in the conventional fermentation, 969 μg/g yield was obtained after 21 days of fermentation [86]. These examples demonstrate the importance of considering the specific microorganism and growth conditions when optimizing light intensity for carotenoid synthesis. Fungi may utilize light to detect high temperatures, UV radiation, and changes in their environment, such as the soil and air interface, which are vital for their survival and reproduction via spore dispersal. Other studies also reported that exposure to blue light is critical in stimulating carotenoid production in other fungi such as *P. blakesleeanus* [87].

### 5.3. Temperature

Temperature can also have an impact on a fungi’s ability to produce carotenoids. Carotenoid production is a complicated process that is influenced by environmental temperature. For example, scientists discovered that the growth rate of *R. glutinis* enhanced as the temperature climbed from 25 to 30 °C but drastically decreased at higher temperatures, and also noted that the carotenoids synthesis is higher at temperatures above 30 °C [88]. Temperature had a slightly different influence on other species, such as *R. mucilaginosa*. Carotenoids synthesis increases at temperatures of 25 and 30 °C. Nevertheless, carotenoid production appeared to decrease in the said specie at temperatures over 30 °C, probably due to yeast enzymatic system denaturation. Yet, another study revealed that when the temperature increased from 10 to 30 °C, both biomass and carotenoid concentration increased. At 23 °C, the mutant strain *R. acheniorum* isolated from milk whey produced the most β-carotene, with a concentration of 262.12 μg/mL [89]. This suggests that for this strain, a slightly cooler temperature is more favorable for carotenoid production [82]. However, for the strain *R. rubra*, there was no significant difference in carotenoid production or biomass growth at temperatures within the range of 20–30 °C. It was concluded that this strain may be less sensitive to temperature variations and may be able to produce carotenoids over a wider range of temperatures [81]. For *R. diobovatum*, the maximum cell growth was observed at 30 °C, indicating that this strain prefers slightly warmer conditions [90]. Finally, a 59-fold increase in β- carotene production (258.8 μg/g) was reported at a temperature of 20 °C compared to 30 °C when *S. cerevisiae* recombinant strain was grown under these temperature conditions [91]. Overall, these findings demonstrate that the optimal temperature for carotenoid production can vary depending on the specific strain of yeast being used. Understanding the temperature preferences of different strains is important for optimizing production conditions and maximizing yields because the complex process of carotenoid production is influenced by the atmospheric conditions, which can alter the enzyme function involved in carotenoid production. The temperature ranges that are optimal for carotenoid production and cell growth are often different. High temperatures are generally beneficial for both processes but at the same time, high temperatures can also denature the enzymes required for carotenoid production.

### 5.4. Carbon and Nitrogen (C/N) Ratio

Nutritional stress, such as limited availability of nitrogen, carbon, or other essential nutrients, has been shown to enhance carotenoid production in many fungal species. This is thought to be because carotenoids serve as antioxidants and protect cells from oxidative stress, which is often induced by nutritional stress. Different yeast strains can respond differently to different carbon sources, while some fungal strains may be better equipped to metabolize certain types of sugars than others, depending on their genetic makeup and metabolic capabilities. Yeast strains such as *Rhodosporidium* and *Phaffia* are able to grow on a variety of sugars found in lignocellulose waste matter [92]. According to previous studies, fungi have the ability to develop and produce carotenoids, which is influenced by the C/N ratio of their growth medium [15]. In particular, it has been discovered that *R. toruloides* and *R. glutinis* prefer a lower C/N ratio of 20:1, but a C/N ratio beyond 30:1 is necessary for lipid biosynthesis [93,94]. These findings imply that increasing the synthesis of certain metabolites by yeast strains can be accomplished by optimizing the C/N ratio of the growth medium.

High C/N ratios (50:1) reduce pigment production because the yeast prioritizes the fatty acid biosynthesis pathway over the mevalonate pathway for carotenoid production. However, a study found that a C/N ratio between 70 and 120, with glucose as the carbon source, positively affected carotenoid synthesis, indicating that yeast can balance the production of both pigments and lipids within a certain range of C/N ratios [95]. In *B. trispora*, the carotenoids synthesis was reported to improve by limiting the nitrogen supply and total C to N ratio of 60. Based on the ingested nitrogen (189.10 mg/g soybean) and carbon source (19.66 mg/g glucose), carotenoid productivity was 983.8 mg/L [96]. It was discovered in our earlier investigation that *M. circinelloides* CBS 277.49 preferred a lower C/N ratio, which led to increased biomass production (15.2 g/L), but this condition was unfavorable for the accumulation of β-carotene [97], while a high C/N, between 40 to 60, as well as yeast/malt extract was reported to enhance astaxanthin production in *X. dendrorhous* [54].

### 5.5. Chemical Stressors

Stress-generating chemicals have been shown to promote the production of carotenoids in fungi [15]. These chemicals can induce stress responses in fungal cells, which can lead to the activation of metabolic pathways that result in increased production of carotenoids. For instance, the carotenoid output of the *X. dendrorhous* or *P. rhodozyma* (PR106) strain was 2.15 times higher than the control after 48 h of exposure to 500 mg/L of titanium dioxide (TiO_2_) stress, and it reached 305.12 mg/L after 72 h [98]. TiO_2_ induced oxidative stress in *X. dendrorhous*, which doubled astaxanthin production without affecting biomass or cell death [99]. The astaxanthin production was found to be doubled (1.30 mg/g) with the administration of H_2_O_2_ at 0 and 24 h to the culture media of *X. dendrorhous*, while volumetric output of 10.4 mg/L was reported. Yet, their research showed that the astaxanthin content increased while β-carotene content decreased noticeably [100].

Similarly, other antioxidants such as butylated hydroxytoluene and *K. rhizophilia* (10%), when fed at the 24th h of fermentation while cultivating *B. trispora*, reported to increase the production of carotenoids up to 7.5-fold, with a maximum accumulation of 793 mg/L carotenoid concertation [101]. Limited calcium in acidic pH was observed to increase the accumulation of carotenes in mucormycota fungi [102]. Magnesium (Mg^2+^) and manganese (Mn^2+^) are essential cofactors in many biochemical reactions, and their availability can affect the synthesis of pigments. For example, it was reported in a study that in the presence of these trace metals, the production of carotenoids was enhanced in *N. intermedia* [103].

## 6. Recent Trends in Biotechnological Production of Fungal Carotenoid

The industrial production of synthetic pigments has increased recently, but due to consumers’ preferences for natural products to avoid any health concerns, the global pigment market has shifted its focus towards the utilization of natural colorants rather than synthetic ones in their products. This strong demand has introduced a new participant to the market: the biotechnological production of carotenoids by native and non-native producers through various strategies. Fungi are considered as potential carotenoid producers as they excel in bioavailability, yield, cost-effectiveness, and simplicity of large-scale cultivation and downstream processing. Fermentation of native carotenogenic fungi *P. blakesleeanus* and *B. trispora* is recognized as a representative method of microbial carotenoid production at the industrial scale [104]. Carotenoid production by carotenogenic fungi is based on the isolation of potential strains, ability to use inexpensive substrates, and the development of an efficient bioprocess for maximum yield, such as optimization of culture media and various fermentation parameters [105]. It has been reported that by mutagenesis and a selection of over-producing strains, carotenogenic fungus can be utilized as a super carotenoid-producing source. A few carotenogenic fungi whose genomic sequence are well recognized allow the identification of the regulatory mechanisms involved in carotenoid biosynthesis, which can then be manipulated to produce strains with specific traits of biotechnological applications [45]. As an alternative, non-carotenogenic fungi are suitable hosts to develop a carotenoid biosynthetic pathway through genetic engineering [106].

### 6.1. Carotenoid Production in Carotenogenic Fungi

To meet the increased demand for industrially important carotenoids, various efforts have been made to improve carotenoid accumulation in native producers such *B. trispora*, *P. blacksealenus*, *H. pluvialisi*, and *X. dendrorhous*. Currently, there are only a few studies aimed at boosting carotenoid production in native producers using metabolic engineering strategies to match the level for commercial-scale production. For instance, *B. trispora* is the sole large-scale industrial approach for β-carotene production at the moment among fungi. This carotenoid was extracted and used as a provitamin A or colorant in crystal form [107]. The industrial procedure for β-carotene production involves the co-culture of *B. trispora* (+) and (−) strains. The best mutants or intersexual heterokaryons with nuclei from both mating strains were able to provide the greatest content of 39 mg/g DCW [108]. Moreover, significant efforts have been made to optimize the culture and development parameters for *B. trispora*, and β-carotene production has reached 78.0 mg/g DCW at lab scales [109]. A mutant strain of *R. glutinis* mutant has been reported to produce 14 mg/L of β-carotene in a shake flask using molasses as the sole nutrient source [110].

*X. dendrorhous* is the only well-known yeast that naturally produces astaxanthin with greater market attraction. However, a very low amount of astaxanthin is produced by wild-type *X. dendrorhous* [111]. For instance, Hu et al. [112] reported 27 mg/L of astaxanthin yield, while Fang et al. [113] found that the astaxanthin yield was only 1.5 mg/L in wild *X. dendrorhous*. In *X. dendrorhous*, overexpressing astaxanthin synthesis-related genes *crtS* and knocking down CYP61 in the mevalonate pathway are two approaches of focus to increase astaxanthin synthesis [111]. CYP61 codes for C-22 sterol desaturases are involved in the negative regulation of the two main rate limiting enzymes (HMGS and HMGR) of the mevalonate pathway. The knock out of this gene increased astaxanthin accumulation by decreasing ergosterol biosynthesis [114,115] and activating the Sterol Regulatory Element-Binding Protein (SREBP) pathway [116]. Gassel et al. [117] achieved 9.7 mg/g DCW of astaxanthin in *X. dendrorhous* by random mutagenesis, overexpression *crtYB*, and *asy*, and selecting optimal growth medium. Their team also obtained 9 mg/g of astaxanthin by combining classical mutagenesis and the concurrent integration of *crtYB*, *crtS*, geranylgeranyl pyrophosphate synthase (*crtE*), and truncated HMG reductase (*hmgR*) [118].

Several studies have explored methods to increase the production of desired metabolites by blocking shared metabolic pathways. By identifying critical enzymes and metabolic bottlenecks, researchers have successfully diverted carbon flux towards astaxanthin production and overexpressed relevant genes, resulting in higher levels of astaxanthin inside the cells [119,120]. Inhibitors of fatty acid synthesis (FAS) and the ergosterol pathway have also been used to improve carotenoid production by redirecting more precursors towards the mevalonate pathway. In a diploid strain of *X. dendrorhous*, researchers developed a promising technique for reducing ergosterol biosynthesis and increasing carbon flux towards astaxanthin biosynthesis by completely deleting the CYP61 gene. This resulted in 1.4 times more astaxanthin production (1.65 mg/L) compared to the parental strain [115]. Moreover, an effective Cre-loxP system has been developed for homologous recombination in *X. dendrorhous*, which overexpresses *crtS* and *crtE*, resulting in astaxanthin content of up to 0.6 mg/g DCW. Using a Cre-loxP system, the transitory expression of the Cre recombinase was managed by a genetically unstable vector independent of episomal plasmids and inducible promoters. This method enables the co-regulation of several genes involved in complicated heterologous astaxanthin metabolic engineering approaches in *X. dendrorhous* [121]. One study aimed to enhance the astaxanthin content in *X. dendrorhous* yeast by utilizing mutagenesis techniques involving UV light and N-methyl-N′-nitro-N-nitroso-guanidine (NTG). The results showed that a UV-irradiated mutant strain of *X. dendrorhous* produced 1.07 mg/g more astaxanthin than the wild strain (0.65 mg/g). The obtained mutant was then subjected to NTG, resulting in a further enhancement of astaxanthin production up to 1.45 mg/g [114].

### 6.2. Carotenoid Production in Non-Carotenogenic Fungi

The microbial production of carotenoids using non-native platforms is appealing due to the many drawbacks associated with natural hosts, such as low abundance and more sophisticated growth requirements, etc. In the past few decades, ketolase genes from *Brevundimonas*, *H. pluvialis*, *Paracoccus*, and *Synechocystis* have been used to obtain ketocarotenoids in various plants [9,122,123]. However, most attempts have resulted in relatively low yields, which were not worthwhile commercially. *P. blakesleeanus* and *B. trispora* have been reported to produce the highest amount of β-carotene, but there are a few downsides connected with the commercial use of these two zygomycetes. For example, shake flask cultivation of *B. trispora* was found to result in reduced carotenoid production, so they should be grown via surface cultivation, and the optimal growth temperature for these fungi should never be above 25 °C [124]. Similarly, *H. pluvialis* is considered a promising natural source for the industrial production of astaxanthin [125] as it can produce a large amount of astaxanthin in their cells up to 5.9% (*w/w*) of the cell dry weight [126,127,128]. Nevertheless, cultivation of this alga at a commercial scale has some unfavorable characteristics such as slow growth and complex morphological transformation, which is associated with the accumulation of astaxanthin in this alga [129]. Moreover, the extraction of astaxanthin from this alga is more hectic due to its accumulation in encysted cells. Because of all these concerns, the production of astaxanthin at an industrial scale from *H. pluvialis* is far away from synthetic astaxanthin in terms of production and cost.

Genetic engineering of non-carotenogenic fungi is considered as another suitable option to obtain a higher production of carotenoids in cells. Despite the high market value of *X. dendrorhous*, the genetic tools for genetic modification for other model yeasts such as *S. cerevisiae* and *Y. lipolytica* are better developed, making them ideal candidates for carotenoid production. A few fungal model organisms of industrial interest for carotenoid synthesis are described in the following sections. Recent progress in various genetic modification strategies to boost carotenoid production in recombinant strains has been described in detail and also summarized in Table 3.

#### 6.2.1. *Y. Lipolytica*

With the advances in metabolic engineering techniques, various microbial strains have been modified for carotenoid production. For example, *Y. lipolytica* has been chosen as the production workhorse by numerous scientists. For lycopene production in this yeast, carotenogenic genes from bacteria *crtB* and *crtI* were incorporated into the genome through CRISPR-Cas9 and the pathway was enhanced by further expression of the genes coding HMG, CrtE, and phosphomevalonate kinase (ERG8), and a total of 3.4 mg/g of lycopene was produced by this strategy in *Y. lipolytica* [130]. The pathway was expanded, and 12.5 mg/g DCW of β-carotene was produced by the same set of fungal genes, except *ERG8*, and by replacing phytoene synthase gene (*crtB*) with the bifunctional *crtYB* [131].

Multiple duplicates of genes for each of the 12 steps of carotenoid biosynthesis and strong promoters were used to construct an efficient biosynthetic pathway that could produce 100 times more β-carotene than the baseline strain. It was shown that the constructed pathway in engineered *Y. lipolytica* produced 4 g/L of β-carotene using a fed-batch fermentation strategy [63]. Larroude et al. [132] genetically engineered the *Y. lipolytica* strain by increasing the lipogenesis, resulting in increased metabolic flux toward the mevalonate pathway. In a single fed-batch experiment, this recombinant strain produced β-carotene up to 6.5 g/L (90 mg/g DCW). One recent study showed the highest β-carotene production 7.6 g/L (159 mg/g DCW) in an engineered strain of *Y. lipolytica* by integrating several copies of 13 genes involved in β-carotene biosynthesis and fed-batch fermentation. The yeast-to-hyphal transition was found with increased carotene production, and the metabolic burden was relieved by deleting *MHY1* and *CLA4* genes to change the mycelial form back to yeast [133]. In one other recent study, the codon-optimized genes *crtI*, *crtE*, and *crtYB* from *X. dendrorhous* were integrated into the chromosome of *Y. lipolytica* for β-carotene production by employing a linearized vector containing strong promoter GPD and achieved 34.5 mg/L of β-carotene production. Then, they carried out the overexpression of key genes of MVA and the fatty acid synthesis pathway and expressed rate-limiting truncated enzyme t*HMGR*. By further increasing the copies of genes related to β-carotene synthesis, production was boosted in the engineered strain up to 117.5 mg/L. Finally, they were able to produce 2695.5 mg/L of β-carotene in fed-batch fermentation by using a 5 L bioreactor [134].

Substrate inhibition of enzymes can act as a major barrier to the production of valuable metabolites in genetically modified microorganisms. Ma et al. [135] showed that the main obstacle in carotenoid production in *Y. lipolytica* was substrate inhibition of lycopene cyclase. Firstly, by structure-guided protein engineering, a modified strain was constructed, with a total loss of substrate inhibition without decreasing enzyme activity. Secondly, they created a GGPP synthase-mediated flux flow restrictor that delays the onset of substrate inhibition by directing metabolic flux away from the inhibitory metabolite while keeping enough metabolic flux for product production. Both methods resulted in the development of an engineered strain with the exclusive ability to produce 39.5 g/L of β-carotene, the highest production reported to date in this yeast [135].

Similarly, *Y. lipolytica* has also been genetically engineered to produce another high-value pigment astaxanthin. Firstly, a platform strain with an enhanced route for β-carotene was developed in small-scale culture, yielding 331 ± 66 mg/L of β-carotene. Then, a different copy number of the β-hydroxylase (*crtZ*) and β-ketolase genes (*bkt*) from algal (*H. pluvialis*) and bacterial *crtW* (*Paracoccus* sp., and *Pantoeaananatis*) sources were inserted into the platform strain for astaxanthin production. It was shown that a modified strain of *Y*. *lipolytica* with algal genes yielded 44 ± 1 mg/L of β-carotene, and the same strain, when grown in controlled bioreactors for seven days on a complex medium with glucose, produced 285.19 mg/L of astaxanthin [67]. Another approach expressed the genes related to astaxanthin biosynthesis individually or in combination in three different compartments of *Y. lipolytica* cells (lipid body, peroxisome, and endoplasmic reticulum) with an aim to understand the potential of subcellular compartments and advance lipid body-based compartmentalized biosynthesis of isoprenoid. Targeting the astaxanthin pathway to subcellular organelles boosted the astaxanthin production and, ultimately, 858 mg/L of astaxanthin was accumulated in the recombinant strain YL17 through fed-batch fermentation [136]. In their most recent study, Zhu et al. [137] optimized the transcriptional expression of *crtW* and *crtZ* by increasing the copy number of the integrated genes into the genome and by using a modular assembly of both enzymes simultaneously, which augmented ability of the modified *Y. lipolytica* strain to convert β-carotene into astaxanthin. Finally, under fed-batch conditions, astaxanthin production increased to 3.3 g/L with a concentration of 41.3 mg/g, the highest level ever recorded in microbial candidates to date [137].

Zeaxanthin is not naturally produced by the carotenoid pathway in *X. dendrorhous* or any other fungi. The gene for astaxanthin biosynthesis (*asy*) was inactivated, and the accumulating β-carotene was transformed into zeaxanthin by the *crtZ* gene from a bacterial source. For the significant conversion of β-carotene to zeaxanthin, reaching up to 5.2 mg/g DCW, it was crucial to incorporate many copies of *crtZ* into the genome. This genetically engineered strain was grown on agricultural waste such as wheat straw hemicellulose hydrolysate to obtain a higher production of zeaxanthin [138]. In one recent study, Xie et al. [139] separately inserted three *crtZ* genes from different sources into the β-carotene-producing *Y. lipolytica* strain for zeaxanthin production. The result showed that *crtz* from the bacteria *P. ananatis* produced the highest titer and content of zeaxanthin, 3.20 mg/g (DCW), by growing on synthetic YNB media.

#### 6.2.2. *S. cerevisiae*

*S. cerevisiae*, being an advanced system, has been widely employed as a host system for carotenoid production by genetic engineering since it has well-developed genetic tools, quick growth, and a wide range of available substrates. Lipid metabolism was one of the areas of focus for carotenoid production. It was observed that inactivating the regulator of lipid droplet size with improved storage capacity is linked to a higher production of lycopene 73.3 mg/g (2.37 g/L) in fermentation with fed-batch mode [140]. A transformant harboring the β-carotene synthesis genes from *X. dendrorhous* was exposed to adaptive laboratory evolution together with oxidative stress, and the selected hyperproducing mutants showed almost 16.4 mg/g DCW of β-carotene synthesis [141]. Transformation of the β-carotene-producing strain with extracellular and cell-bound lipases boosted its ability to utilize waste olive oil as a growth substrate. The lipid content of the cell was increased by supplying olive oil in media as a supplement, which provided a significant amount of acetyl-CoA, which led to 46.5 mg/g DCW (477.9 mg/L) of β-carotene [142]. To boost the accumulation capacity of β-carotene to produce the *S. cerevisiae* strain, Zhao et al. [143] modified various lipid metabolic pathways and inspected the connection between lipid components and the accumulation of β-carotene. They found a 1.5-fold increase in β-carotene content by overexpressing the genes responsible for sterol ester synthesis such as *ARE1* and *ARE2*. A two-fold increase in β-carotene yield was also seen after the deletion of the phosphatidate phosphatase genes (*PAH1*, *DPP1*, and *LPP1*). Finally, by combining these two approaches, the resulting recombinant strain YZ30 produced 8.98 mg/g DCW of β-carotene [143]. In one study, a xylose-fermenting strain of *S. cerevisiae* was genetically modified for β-carotene production by introducing related genes such as *crtYB*, *crtI*, and *crtE* from *X. dendrorhous*. The constructed recombinant strain produced 772.8 mg/L of β-carotene using xylose as substrate in fed-batch fermentation [144].

Zhou et al. [145] genetically engineered the *S. cerevisiae* for astaxanthin production by the overexpression of *crtZ* and *bkt* from *H. pluvialis* and finally obtained 4.7 mg/g of astaxanthin per DCW in shake flask fermentation. Furthermore, *tHMG1* (truncated), *crtI*, and *crtYB* were overexpressed along with a positive mutant strain of GGPP synthase, and a final yield of 8.10 mg/g (47.18 mg/L) of astaxanthin was obtained [146]. Through metabolic engineering, it was possible to compare the activity of *crtZ* from *Agrobacterium aurantiacum* and c*rtZ* from *Alcaligenes* spp. in the *S. cerevisiae* for astaxanthin production. To increase astaxanthin production by *S. cerevisiae*, Jin et al. [147] carried out heterologous gene expression of β-carotene ketolase (*crtW*) from *Brevundimonas vesicularis* and *crtZ* from *A. aurantiacum*. They also applied mutagenesis via atmospheric and room temperature plasma ARTP and attained an astaxanthin concentration of 13.8 mg/g DCW (217.9 mg/L) following fermentation in a 5 L fermenter.

According to related research, the key problems limiting the exogenous astaxanthin accumulation were the lower efficiency of β-carotene ketolation and hydroxylation, along with the detrimental impact of astaxanthin accumulation on cell development in *S. cerevisiae*. To address these issues, Zhou et al. [148] carried out directed evolution of β-carotene ketolase and hydroxylase as well as the development of a temperature-responsive regulatory system to improve astaxanthin synthesis in *S. cerevisiae*. This temperature-regulation system based on Gal4M9 was developed to separate the production stage from the growth stage in *S. cerevisiae* in order to achieve high cell density astaxanthin fermentation, resulting in an astaxanthin titer of 235 mg/L. The introduction of the astaxanthin synthesis pathway along with ARTP mutagenesis and adaptive evolution of a recombinant *S. cerevisiae* strain led to the generation of a super astaxanthin producer, with an astaxanthin yield of 404.78 mg/L in a 5 L bioreactor [149]. Li et al. [150] examined a library of genes involved in the metabolism of lipids in *S. cerevisiae* using the trifunctional CRISPR technology and discovered *opi3* and *hrd1* as novel engineering goals for better astaxanthin production by moderately, rather than excessively, upregulating the synthesis of lipids. After lipid engineering, the astaxanthin content was 9.79 mg/g DCW; however, by balanced expression of β-carotene hydroxylase and ketolase, it was increased to 10.21 mg/g DCW [150].

#### 6.2.3. *M. circinelloides*

*M. circinelloides* is a β-carotene-producing Mucormycota fungus and is reported as one of the model microbial systems to study the biosynthesis of carotenoids in fungi due to the availability of genetic tools and genomic sequences that can help in the construction of a high β-carotene-producing strain. Moreover, it is an attractive target of biotechnological developments due to its dimorphic nature [151] and the availability of efficient transformation tools, which improve carotenoid production [152]. *M. circinelloides* is reported as a naturally competent organism in comparison to other related species or genera, and has also been proven as an ideal host for the heterologous expression of genes [69,153].

Zhang et al. [45] reported that disrupting *crgA* resulted in the over-accumulation of β-carotene in mutant *M. circinelloides* strain up to 4 mg/g. They tried to understand the regulation of carotenoid biosynthesis and develop candidate strains for the biotechnological production of β-carotene by combining classical forward and modern reverse genetic approaches. Moreover, Nagy et al. [154] developed a plasmid-free CRISPR-Cas9 system to genetically modify Mucorales. The defined technique provides a quick yet reliable tool to produce *M. circinelloides* mutants that are mitotically stable through the targeted integration of the desired genes. By deleting two distinct genes from *M. circinelloides*, *carB*, which encodes phytoene dehydrogenase, and *hmgR2*, which encodes HMG-CoA reductase, the effectiveness of the technique for the non-homologous end joining NHEJ and HDR was evaluated. Stable gene disruption mutants were produced as a result of NHEJ and HDR, with carotenoid content of the *hmgR2* mutant (457 ± 74 μg/g DCW [154].

Csernetics et al. [32] expressed the *crtZ* gene of *Paracoccus* in canthaxanthin-producing mutants of *M. circinelloides* to promote astaxanthin production. They obtained a maximum of 443 ± 71 μg/g of canthaxanthin and 14 μg/g of astaxanthin in one mutant, and with further medium optimization, they obtained astaxanthin content up to 35 μg/g in a modified strain. Csernetics et al. [31] also introduced two genes *crtR* and *crtS* in *M. circinelloides* from X. *dendrorhous* for the conversion of β-carotene to astaxanthin. After arduous genetic modification, a maximum of 190 μg/g of canthaxanthin was produced in a mutant *M. circinelloides* strain. However, the canthaxanthin and astaxanthin yield was still too low compared to its native producers. Similarly, a canthaxanthin-producing *M. circinelloides* strain was constructed in a recent study by introducing codon-optimized *bkt* from *H. pluvialis* into the genome of the fungus by employing promoter *zrt1*. In their study, the *crgA* gene was disrupted, resulting in a significant increase in β-carotene production, which acts as the substrate for canthaxanthin through the enzymatic action of ketolase. The genetically modified strain was accumulated (576 ± 28 μg/g) from canthaxanthin, which is the highest concentration reported in *Mucor* to date [69]. However, in this fungal model, the production of carotenoids is still far beyond industrial-level production. A comprehensive strategy is required in future to explore its maximum capacity for the heterologous production of xanthophylls.

**Table 3:** Carotenoid production by native producers and engineered non-carotenogenic using biotechnological approaches.

Microorganism	Carotenoid	Genotype	Fermentation	Productivity	Reference
*B. trispora*	β-carotene	Wild type	Shake flask	39 mg/g DW	[108]
			optimized culture and development conditions	78.0 mg/g	[109]
*R. glutinis*	β-carotene	Wild type	Shake flask using molasses	14 mg/L	[110]
*X. dendrorhous*	Astaxanthin	Random mutagenesis, overexpression of *crtYB* and *asy*	Optimal growth medium Bioreactor	9.7 mg/g DCW	[117]
	Astaxanthin	CYP61 deletion	Sakaguchi flask	1.65 mg/L	[115]
	Astaxanthin	*Cre-loxP*, overexpressing *crtS* and *crtE*	Liquid YM medium	0.6 mg/g	[121]
	Astaxanthin	UV-radiated mutant, NTG	Liquid YM medium	1.07 mg/g, 1.45 mg/g	[155]
*Y. lipolytica*	Lycopene	Bacterial *crtB* and *crtI* integration	YPD, Shake flask	3.4 mg/g	[130]
	β-carotene	Fungal *crtBY* and *crtI*	YPD, Shake flask	12.5 mg/g	[131]
	β-carotene	Multiple gene integration	Fed-batch fermentation	4 g/L	[63]
	β-carotene	Golden Gate DNA assembly	Single fed-batch experiment	6.5 g/L (90 mg/g DCW	[132]
	β-carotene	Integrating multiple copies of 13 genes	Fed-batch fermentation.	7.6 g/L (159 mg/g)	[133]
	β-carotene	Yeast *crtI*, *crtE*, and *crtYB*, overexpression of key genes of MVA and FAS pathway	Fed-batch fermentation by using 5 L fermenter	2695.5 mg/L	[134]
	β-carotene	Structure-guided protein engineering	5 L fermenter	39.5 g/L	[135]
	Astaxanthin	Algal β-ketolase genes	Controlled bioreactor with glucose	285.19 mg/L	[67]
	Astaxanthin	β-ketolase and hydroxylase genes	Fed-batch fermentation	858 mg/L	[136]
	Astaxanthin	Modular enzyme assembly of *CrtW* and *CrtZ*	Fed-batch fermentation	3.3 g/L	[137]
	Zeaxanthin	Bacterial multiple copies of copies of *crtZ*	Wheat straw hemicellulose hydrolysate	5.2 mg/g DCW	[138]
	Zeaxanthin	Bacterial *crtZ*	Synthetic YNB medium	3.20 mg/g	[139]
***S. cerevisiae***	Lycopene	Over expression of *OLE1*.	Fed-batch fermentation	73.3 mg/g (2.37 g/L)	[140]
	β-carotene	Adaptive laboratory evolution of β-carotene synthase gene	Oxidative stress	16.4 mg/g DCW	[141].
	β-carotene	Modification of extracellular and cell-bound lipases	Grown on olive mill waste oil	46.5 mg/g DCW (477.9 mg/L)	[142]
	β-carotene	Overexpression *ARE1* and *ARE2 and* deletion of (*PAH1*, *DPP1*, and *LPP1*).	Shake flask fermentation	8.98 mg/g DCW	[143].
	β-carotene	Yeast genes *crtYB*, *crtI*, and *crtE*	Xylose as substrate in fed-batch fermentation	772.8 mg/L	[144]
	Astaxanthin	Overexpression of algal *crtZ*) and *bkt*)	Shake flask fermentation	4.7 mg/g DCW	[145]
	Astaxanthin	Over expression of *tHMG1*(truncated), *crtI*, and *crtYB* along with a positive mutant of GGPP synthase	Shake flask fermentation	8.10 mg/g (47.18 mg/L)	[146]
	Astaxanthin	*crtW* and *crtZ* along with mutagenesis using ARTP	Batch fermentation in 5 L fermenter.	13.8 mg/g DCW (217.9 mg/L)	[147]
	Astaxanthin	Directed evolution of β-carotene hydroxylase and ketolase	2 stage high density fermentation	235 mg/L	[148]
	Astaxanthin	Upregulation of *opi3* and *hrd1* using trifunctional CRISPR Balancing expression of *bkt* and *crtZ*	Fed-batch fermentation.	9.79 mg/g DCW 10.21 mg/g DCW	[150]
*M. circinelloides*	β-carotene	Disruption of *crgA*	Shake flask fermentation	4 mg/g DCW	[45]
	Canthaxanthin, Astaxanthin	Bacterial *crtZ* gene	Shake flask fermentation	443 ±71 μg/g 35 μg/g DCW	[32]
	Canthaxanthin	Yeast *crtR* and *crtS*	Solid media cultivation	190 μg/g DCW	[31]
	Canthaxanthin	Algal *bkt*	1.5 L bioreactor	576 ± 28 μg/g DCW	[69]

## 7. Carotenoid Extraction: Methods, Considerations, and Downstream Impacts on Cost

The extraction of lipophilic carotenoids, particularly astaxanthin, requires organic solvents. The polarity of the carotenoid is important in determining the use of solvent, and it can be difficult to separate carotenoids with various polarity from the same system [156]. On the other hand, the microbial carotenoids extraction process is important for industrial applications. To recover and process carotenoids, a series of downstream operations is required due to their intracellular nature.

**Figure 3:**
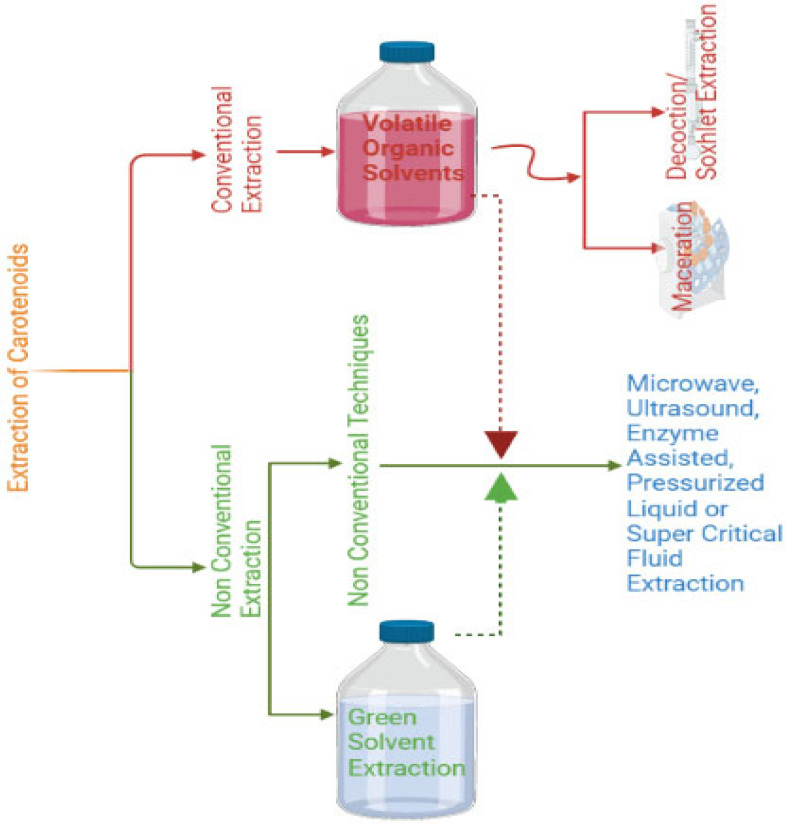
Various carotenoid extraction methods.

Traditional filtration or centrifugation techniques are used in the initial stage of extraction to separate the supernatant from the cellular biomass, which contains the intracellular carotenoids. The release of intracellular carotenoids is then facilitated by various physical, chemical, and/or biological techniques used to disrupt cells. The intracellular carotenoids are then drawn out and isolated from the cell debris. The steps of cell disruption and extraction can be completed independently or as a single process. For instance, a soxhlet extraction and a chemical pre-treatment of the cells can be coupled. Certain carotenoids can then be saponified and isolated during later phases of processing. Therefore, in order to effectively extract intracellular carotenoids from robust cells, more vigorous extraction techniques are required, such as cryogenic grinding or the application of chemical agents such as an acid/base, surfactant, volatile organic solvents (VOCs), or a mixture of these agents [157]. VOC-based extraction methods offer high extraction yields; however, the use of these chemical agents can be a potential hazard to the health and the environment [158]. In recent years, researchers have explored alternative and efficient techniques to overcome these concerns. One such approach is substituting VOCs with more environmentally friendly, biocompatible, and less harmful solvents, such as green solvent extraction, and also utilizing these in non-conventional techniques such as enzymatic-assisted or supercritical fluid extraction, etc. [159]. The techniques for carotenoid extraction are summarized in Figure 3.

### 7.1. Conventional Carotenoid Extraction and the Impact of VOCs

VOCs are widely used in traditional extraction techniques, such as atmospheric liquid extraction employing maceration or soxhlet. Yet, unconventional techniques such as enzyme- or ultrasound-assisted extraction (EAE) can also make use of VOCs. Non-polar solvents such as tetrahydrofuran (THF), petroleum ether, or hexane are excellent options for extracting non-polar carotenoids since they may efficiently dissolve carotenoid molecules and damage cell walls and membranes. These solvents are particularly intriguing since they are based on the extractant and they can enter microbial cells to solubilize intracellular carotenoids. On the other hand, polar solvents, including dimethyl sulfoxide (DMSO), acetone, ethanol, and ethyl acetate, are more effective for extracting polar carotenoids [157]. The efficacy of various VOC combinations were evaluated in conjunction with liquid nitrogen, using a traditional technique that involves VOC atmospheric liquid extraction and multiple macerations of *Sporidiobolus salmonicolor* CBS 2636 cellular biomass. And the study’s findings revealed that total carotenoids concentration reached its highest (253.8 μg/g) by employing liquid nitrogen before cell disruption with DMSO, followed by a liquid extraction by utilizing acetone and methanol with a 7:3 *v*/*v* ratio [160].

This finding is particularly noteworthy as it indicates that a specific combination of VOCs can significantly impact the recovery of carotene-based pigments from cellular biomass. The process of lyophilization and liquid extraction proved to be a successful way to extract total carotenoid content from *R. glutinis* cells when lyophilized *R. glutinis* cells were added with the organic solvent mixtures directly. Hexane had the lowest extraction capacity (0.19 mg/g), according to the study’s findings, whereas petroleum ether and DMSO solvents had the highest recovery yields (0.23 mg/g and 0.24 g/g, respectively) [161]. These investigations show that the choice of solvent has a substantial impact on the extraction of carotenoids from biomass and that lyophilization can increase the extraction process effectiveness.

Similarly, another traditional method such as soxhlet extraction uses heat along with solvents to extract compounds such as carotenoids, but it is time-consuming and costly. In comparison, accelerated solvent extraction (ASE) is a faster and more cost-effective alternative that can selectively extract carotenoids from pressed palm fiber. The ASE method exhibited greater selectivity and generated a higher yield of carotenoids compared to soxhlet or percolation methods. At a manufacturing cost of USD39.1/kg, ASE also proved to be the most economical approach of the three, as opposed to USD98.1/kg for soxhlet and USD48.9/kg for percolation. Soxhlet extraction can be replaced by ASE as a superior substitute for the extensive extraction of carotenoids [162].

### 7.2. Greener Solvent Extraction

The conventional use of volatile organic compounds (VOCs) in carotenoid extraction techniques poses a potential threat to the environment. To increase sustainability and reduce environmental impact, researchers have been exploring the use of green solvents such as supercritical CO_2_, subcritical H_2_O, ionic liquids (ILs), or other solvents obtained from renewable and non-renewable sources that are non-toxic and biodegradable [163]. Supercritical fluids are the first generation of green solvents, which possess both the properties of liquid and gas at a certain temperature and pressure, known as critical temperature and pressure. Comparatively, a new class of green solvents include the ILs, which can be defined as the liquid salt mixtures in which individual ionic components bind with one another through ionic bonds and become liquid at room temperature, and their vapor pressure is negligible (<1 Pa) [164]. Deep eutectic solvents (DES) are considered as the fourth generation ILs and are combinations of both ionic and non-ionic molecules that form hydrogen bond interactions as opposed to ILs, which are constituted of a cation and an anion. DES are inexpensive, biodegradable, and simple to make by combining the hydrogen bond donor (HBD) and hydrogen bond accepter (HBA) [165]. According to an investigation by Yara-Varon et al., 2-methyltetrahydrofuran and cyclopentyl methyl ether were efficient alternatives to hexane for the separation of carotenoids from microbiological sources, with higher yields obtained using these green solvents [159], while for astaxanthin extraction from *X. dendrorhous*, ethanol was reported as the most efficient solvent, with a yield of 114.284 μg/g of astaxanthin after the cell disruption treatment with acid and ultrasound [166]. Additionally, surfactant aids have been identified to be effective in breaking down cell membranes and extracting carotenoids [167].

Ionic liquids (ILs) are green solvents that are environmentally friendly and non-flammable. Ethylammonium caproate is a biocompatible proton IL that can dissolve the cell wall, and it has advantages over imidazole-based ILs as it is inexpensive, easy to synthesize, and has good biocompatibility. Combining multiple methods can improve the extraction efficiency of carotenoids. ILs, HCl, and high-pressure micro-fluidization treatment were found to be the most efficient in cell disruption, with more than 80% astaxanthin recovery [168]. A new extraction system that used a CO_2_-based alkyl carbamate ILs was developed to extract astaxanthin from *H. pluvialis*, with dimethyl ammonium dimethyl carbamate as the best-performing IL, with 27.99 mg/g of astaxanthin recovery, under optimal extraction conditions [169]. This technique can also be utilized for the extraction carotenoids from fungal species. Recently, researchers achieved approximately 40% *w*/*w* recovery of astaxanthin and β-carotene from the *X. dendrorhous* by using cholinium octanoate-based IL [170]. This suggests that VOCs in carotenoid extraction techniques can be reduced for sustainability and to lessen their environmental impact.

### 7.3. Non-Conventional Techniques for Carotenoid Extraction

Non-conventional techniques for carotenoid extraction offer several advantages over conventional methods, including improved efficiency, reduced processing time, and lower solvent consumption.

#### 7.3.1. Super Critical Fluid Extraction

Recent research has focused on developing alternative methods to traditional solvent extraction. One such method gaining popularity is supercritical fluid extraction (SFE), which utilizes safe solvents such as carbon dioxide and water to extract valuable bioactive compounds. The SFE offers higher yields, reduced extraction times, improved selectivity, and lower environmental impact compared to traditional methods. The SFE is considered a reliable and sustainable method for carotenoid extraction, with higher diffusibility and permeability, leading to faster and more efficient extraction [171].

When the effectiveness of SFE was compared with traditional liquid extraction (acetone-based) for extracting astaxanthin from *X. dendrorhous*, the findings showed that CO_2_-based SFE at 500 bar pressure and 40 °C had 3.6 times the amount of astaxanthin as acetone-based extraction at the same temperature, and a 13-fold increase when the temperature was raised to 60 °C. The highest yield of 90% was achieved using CO_2_ (50 g), while temperature was a key factor in the SFE process [172], although, SFE is a sustainable method for extracting pure thermolabile compounds such as carotenoids. However, because of their high molecular weight, carotenoids occasionally experience solubility reductions that may impair the effectiveness of the extraction procedure. Modifiers such as methanol and ethanol are added to CO_2_ to increase the extraction yield in order to solve this problem [173]. For example, the efficiency of extracting polar carotenoids, particularly xanthophylls, is often inadequate. Organic modifiers, such as ethanol, can enhance analytes’ solubility and minimize their contact with the sample matrix, resulting in the release of analytes. Adding ethanol as an organic modifier to SFE increased the extraction efficiency of astaxanthin from *H. pluvialis* by improving the bioavailability of the compound [174]. Therefore, adding an organic modifier to SFE can be a feasible approach to improve the extraction yield of polar carotenoids. Another possible way to enhance the yield is adjusting the polarity of CO_2_ by manipulating pressure and temperature to match the polarity of the targeted carotenoid, which could result in higher extraction yields. Increasing the CO_2_ flow rate and pressure while decreasing the moisture content also improved the carotenoid yield. However, the use of co-solvents at 2% and 5% concentrations, such as hexane/isopropanol, was not effective in enhancing carotenoid yields due to the high lipid content in pink shrimp, which affected the selectivity towards lipid components, leading to lower carotenoid yields. The study demonstrated the potential of SC-CO_2_ extraction for achieving high yields of carotenoids in the sample under optimized conditions. The authors suggested that increasing the CO_2_ flow rate could improve mass transfer rates, thereby increasing the concentration gradient between the sample and solvent phases through the convection mechanism [175].

#### 7.3.2. Pressurized Liquid Extraction (PLE)

PLE can enhance the efficiency of carotenoid extraction by improving cell permeability, promoting intermolecular interactions, and increasing the penetration of extracting solvents. The use of higher temperatures reduces the viscosity of the solvents and facilitates their diffusion into the sample matrix. Furthermore, pressure-induced denaturation of carotenoid-binding proteins might enhance the effectiveness of carotenoid extraction [176]. A study has shown that the pressure during the extraction process can affect carotenoid yield. High-pressure homogenization is a safe and efficient cell wall-breaking method for carotenoid extraction from *S. pararoseus*, with the highest carotenoid yield (82.5%) being obtained from it compared to other cell wall-breaking techniques [177]. Similarly, Martínez et al. recovered 25 mg/L of carotenoids from *R. glutinis* by optimizing the pressure and ultrasound-assisted extraction [178]. Various studies have reported optimal PLE conditions for extracting carotenoids from different sources, such as microalgae and fruits. For example, The frozen biomass of the microalga *N. oleoabundans* yielded the maximum carotenoids recovery at 100 °C, 20 min of extraction time, and 103 bar of pressure, with 100% ethanol as the extractant [179]. Accelerated solvent extraction (ASE) has also been found to offer advantages over conventional solvent extraction processes [180].

In summary, PLE is a promising method for extracting carotenoids from various sources, including fungi, fruits, and microalgae. The use of optimized extraction conditions, such as temperature, pressure, and solvent composition, can significantly enhance the extraction efficiency of carotenoids.

#### 7.3.3. Enzyme, Ultrasound, Microwave, and Electric Field Assisted Extraction of Carotenoids

Hydrolytic enzymes are used in enzyme-assisted extraction (EAE) techniques to disrupt the structural integrity of cell walls, allowing for better extraction yields of intracellular materials. As the traditional solvent extraction method may affect the stability of carotenoids due to the heat generated during the process, so EAE has been introduced to improve stability. Studies have shown that enzyme-assisted extraction can enhance the stability of astaxanthin. Enzymatic extraction has several advantages, such as being environmentally friendly, renewable, and specificity [157,181]. However, enzymes are expensive and require optimal operating conditions, so microwave and ultrasonic-assisted extraction technologies have also been developed to overcome low yield and high extraction costs. Traditional single microwave or ultrasonic extraction methods may not be sufficient for large-scale production, leading to the development of various combined extraction technologies. For instance, a recent study reported the use of lactic acid to disintegrate cell clusters in *X. dendrorhous*, followed by efficient astaxanthin extraction using ultrasonic technology [182]. For ultrasound-assisted extraction, the recovery of 90% astaxanthin from *X. dendrorhous* was achieved by using three-molar lactic acid [166], while the greatest recovery and extraction rates of astaxanthin achieved so far were 95.08 ± 3.02% and 99.74 ± 0.05%, respectively, under ideal operating conditions [183]. The use of ultrasound in combination with enzyme-assisted extraction can further enhance extraction efficiency by promoting the penetration of the enzyme into the microbial cell wall and facilitating the release of the target compounds.

Moreover, the method of pulsed electric field (PEF) technology involves the application of short high-voltage pulses to a biological material placed between two electrodes, enabling the extraction of intracellular carotenoids without any thermal intervention. This approach induces the increased permeability of the cytoplasmic membrane, which is known as electroporation. The level of membrane permeability is dictated by the intensity of the electric field, which can lead to either reversible or irreversible changes. The application of PEF technology has shown great promise as an energy-efficient and non-thermal method for extracting carotenoids. For example, researchers successfully extract 70% of astaxanthin (84% trans astaxanthin) by using PEF with aqueous incubation and ethanol as an extractant [184]. However, to optimize the process, the duration of the pulses and the intensity of the electric field need to be adjusted for each sample matrix, as changes in the electrical conductivity and texture of tissue can affect these parameters. Therefore, it is important to carefully optimize the process to ensure effective extraction [185].

## 8. Exploring the Potential of Fungal Pigments for Industrial Applications: Challenges in Commercialization

Carotenoids, a class of naturally occurring pigments present in plants and animals, as well as microorganisms such as fungi and algae, include the pigments canthaxanthin, astaxanthin, zeaxanthin, and lycopene, etc. Additionally, carotenoids provide a variety of health advantages, such as antioxidant and anti-inflammatory properties as well as cancer prevention. For example, red carotenoid canthaxanthin is used as a nutritional supplement and food ingredient. Numerous fungi, including *X. dendrorhous* and *B. trispora*, can synthesize canthaxanthin [186,187], while astaxanthin, a red carotenoid, has been found to provide a range of health advantages, including protection against cancer, cardiovascular disease, and age-related macular degeneration [188]. The retina of the eye contains zeaxanthin, a yellow carotenoid. Zeaxanthin may help lower the risk of age-related macular degeneration since it has been demonstrated to shield the retina from harm caused by blue light [189]. *R. glutinis*, *R. rubra*, and *B. trispora* are capable of producing zeaxanthin [190]. Fungi is also capable of producing lycopene, a red carotenoid, which has numerous health advantages, including defense against prostate cancer, cardiovascular disease, and cancer [191].

Fungi-produced carotenoids have a number of benefits, including the ability to grow well under controlled conditions and the ability to be genetically modified, enabling the manufacture enormous amounts of carotenoids. They possess great potential for use in multiple industries, including food, cosmetics, textiles, paper production, as well as agrochemical and pharmaceutical applications [103]. Despite this wide range of potential applications, only a few fungal pigments are currently being produced and sold at an industrial scale. Consequently, there exists a great opportunity to overcome this gap between laboratory research and commercialization.

Fermentation is a low-cost and scalable process for generating carotenoids. It is also a sustainable solution because it does not rely on land or water resources. In the future, the fermentation approach is projected to remain the major method for manufacturing carotenoids, because fungi can be genetically modified and can be grown in controlled settings to produce a large number of carotenoids. Despite this, only a few firms dominate the industrial manufacture of carotenoids, notably BASF, DSM, and Cyanotech. Furthermore, in 2021, the market for carotenoids was estimated to be worth USD 1.8 billion, and by 2031, it is anticipated to be worth USD 2.7 billion, growing at a CAGR rate of 3.9% (https://www.alliedmarketresearch.com/carotenoids-market (accessed on 15 April 2023)).

Despite their numerous applications, the production and use of fungal pigments at an industrial scale are still challenging due to several factors. One of the main challenges in the production of fungal pigments is low yield. To improve yield, optimizing the fermentation process and developing high-yield fungal strains are potential solutions. Genetic engineering techniques can be used to enhance the production of fungal pigments and reduce production time. Additionally, maintaining optimal fermentation conditions, such as temperature, pH, and nutrient availability, can be difficult when scaling up production. The use of automated monitoring and control systems can help maintain optimal fermentation conditions and increase production efficiency. Downstream processing, including extraction and purification, is also challenging and time-consuming due to the complex mixture of fungal pigments. Developing more efficient and selective extraction and purification methods, such as chromatography and membrane filtration, can increase the purity and yield of fungal pigments. These methods can also reduce processing time. Furthermore, fungal pigments are often sensitive to light, temperature, and pH, making their stability and color intensity a challenge in industrial applications. Encapsulating fungal pigments within food-grade materials or using innovative packaging technologies can improve their stability and prolong their shelf life. Cost is another significant challenge in the production of fungal pigments at an industrial scale due to the high costs of raw materials, equipment, and skilled labor required for fermentation and downstream processing. Utilizing cheaper substrates and optimizing process parameters with genetic modifications can help reduce the overall production costs and make fungal pigments more commercially viable. Lastly, regulatory challenges, including obtaining necessary approvals and certifications, make it challenging to use fungal pigments in industrial applications. Establishing partnerships with regulatory agencies and complying with safety standards can facilitate the approval process for using fungal pigments in various industrial applications.

Despite these challenges, the potential benefits of using natural fungal pigments make them a promising area of research and development. Therefore, there is a need for continued research to address these challenges and improve the production and use of fungal pigments at an industrial scale.

## 9. Conclusions

Fungal carotenoids have applications in various industrial and medical fields. Naturally-derived carotenoids have gained approval for human consumption, and the antioxidant activity of astaxanthin produced by biological means is reported to be higher compared to chemical synthesis. Despite this wide range of potential applications, only a few fungal pigments are currently being produced and sold at an industrial scale due to lower production yield with high costs and a lack of genetic tools. In this review, we highlighted a few fungal carotenoids with commercial significance and described the latest research being conducted to genetically modify carotenogenic and non-carotenogenic fungi to enhance carotenoid production in model chassis, with an aim to meet the demand of industrial production. After successful production in microbial cells, the extraction of carotenoids is another important parameter to obtain maximum yield. This review summarized various conventional and non-conventional methods to extract the maximum carotenoids from fungal and yeast cells. However, yield from fungal sources is still too low for industrialization. Therefore, more productive and robust candidates need to be screened, the the cultivation conditions need to modulated, and highly efficient genetic manipulation tools and techniques need to be developed.

## Figures and Tables

**Table 2:** Main carotenoids produced by selected model fungal species.

Fungal Species	Carotenoids	Reference
Ascomycota:		
*Fusarium* species	Neurosporaxanthin and torulene	[35,36,37]
*Neurospora crassa*		
Basidiomycota:		
*Xanthophyllomyce dendrorhous*	Astaxanthin and β-carotene	[38,39]
*Rhodotorula glutinis*	Torulene, torularhodin and β-carotene	[40]
*Cantharellus* species	Canthaxanthin	[41]
*Rhodotorula* and *Sporobolomyces roseus*	Torulene, torularhodin, and β-carotene	[42]
Mucoromycota:		
*Blakeslea trispora*, *Phycomyces blakesleanus*	Lycopene and β-carotene	[43,44]
*Mucor circinelloides*	β-carotene	[13]

## Data Availability

Not applicable.
